# Editorial: ICAR2024 highlights

**DOI:** 10.3389/fpls.2026.1926580

**Published:** 2026-07-16

**Authors:** Rezwan Tanvir, Yongjian Qiu, Cankui Zhang, Ling Li

**Affiliations:** 1 Department of Biological Sciences, Mississippi State University, Mississippi State, MS, United States; 2 Department of Biology, University of Mississippi, University, MS, United States; 3 Department of Agronomy and Center for Plant Biology, Purdue University, West Lafayette, IN, United States

**Keywords:** *Arabidopsis thaliana*, gene regulatory networks, hormone signaling, live-cell imaging, model-to-crop translation, multi-omics, plant–environment interactions

The International Conference on Arabidopsis Research (ICAR) has long served as a central forum for presenting new discoveries, exchanging ideas, and building collaborations across the Arabidopsis research community. As a premier model system for plant biology, *Arabidopsis thaliana* has provided foundational insight into the molecular mechanisms that regulate plant growth, development, metabolism, and environmental responses (Koornneef and Meinke, 2010; Woodward and Bartel, 2018). Equally important, the extensive genetic, genomic, and molecular resources developed in Arabidopsis have established conceptual and experimental frameworks that continue to inform translational research in agriculturally important species (Yaschenko et al., 2025; Brady et al., 2025).

The Research Topic “ICAR2024 Highlights” brings together a focused collection of studies that reflect the breadth and continuing vitality of Arabidopsis-centered research. Rather than serving only as a record of conference presentations, this Research Topic highlights work that advances mechanistic understanding, develops enabling technologies, and connects model-system discoveries to crop improvement. The four contributions span gene regulation, hormone-mediated immunity, live-cell imaging technology, and drought-related trait discovery in soybean, while sharing a common emphasis on experimental rigor and biological relevance.

Two contributions examine how genes and signaling networks shape plant physiology and defense. Tanvir et al. investigate the function of *Taxonomically Restricted QQS Associated 1* (*TRQA1*), a Brassicaceae-restricted gene identified through its reciprocal expression relationship with the Arabidopsis orphan gene *Qua-Quine Starch* (*QQS*). *QQS* is known to increase protein content and reduce starch accumulation across plant species. In contrast, altered *TRQA1* expression produced the opposite metabolic pattern: *TRQA1* overexpression increased starch accumulation and reduced protein content, whereas *TRQA1* suppression or T-DNA insertion reduced starch and increased protein. Using transgenic and mutant lines, promoter–reporter analysis, subcellular localization, and AlphaFold2-Multimer modeling, the authors propose that *TRQA1* may influence carbon and nitrogen allocation through a regulatory framework involving *QQS*, *MYB103*, and *NF-YC4*. This study expands the functional landscape of taxonomically restricted genes and illustrates how lineage-restricted genes can become integrated into broader metabolic regulatory networks.

Li et al. address hormone crosstalk at the plant–pathogen interface by examining the role of melatonin in Arabidopsis stomatal immunity. Stomata are a major entry point for bacterial pathogens, and their closure represents an early defense response. Using melatonin biosynthesis and receptor mutants, stomatal movement assays, single-cell-type metabolomics, and complementary multi-omics analyses, the authors show that melatonin is required for maintaining stomatal closure during *Pseudomonas syringae* infection. Their data indicate that melatonin suppresses jasmonic acid biosynthesis, thereby reducing antagonism with salicylic acid signaling and promoting bacterial resistance. The study further implicates the melatonin receptor PMTR1 and ROS/NO-associated signaling in coordinating guard-cell immune responses. Together, these findings provide a detailed view of how melatonin modulates hormone balance and defense signaling during the early stages of pathogen interaction.

Methodological innovation is also essential for progress in plant biology, and Bajracharya et al. address a practical limitation in live-cell imaging of thermal responses. Precise temperature control on the microscope stage is critical for studying heat stress, heat shock responses, and thermomorphogenesis, but commercial systems can be costly and inaccessible to many laboratories. The authors developed two affordable heating devices for confocal microscopy: an aluminum heat plate and a wireless mini-heater. The heat plate supports controlled moderate warming, whereas the wireless mini-heater enables rapid and precise heating directly at the sample slide and can reach temperatures suitable for severe heat-stress and heat-shock studies. The devices were validated by real-time imaging of heat shock protein accumulation and stress granule dynamics in Arabidopsis seedlings and protoplasts. By lowering technical and financial barriers, this work provides accessible tools for dynamic imaging of plant thermal biology.

Finally, Parajuli et al. demonstrate how knowledge gained from Arabidopsis continues to facilitate crop trait discovery. The authors conducted genome-wide association studies in soybean for two drought-related traits: a visible leaf-flipping phenotype and transpiration-related measurements obtained with wearable plant sensors. By integrating GWAS, deep short-read sequencing, transcriptomic information, *cis*-element analysis, and functional inference from homologous genes, they narrowed 67 candidates to seven putative drought-tolerance genes. Several candidates were interpreted in part through knowledge from Arabidopsis homologs, underscoring the continued value of Arabidopsis as a reference for functional annotation in crops. The use of wearable sensors to quantify transpiration-associated traits also illustrates how emerging phenotyping technologies can increase the resolution of association studies in complex crop genomes.

Taken together, the studies in this Research Topic capture several themes that were prominent at ICAR2024: the dissection of gene regulatory and hormone-signaling networks, the integration of multi-omics approaches, the development of accessible experimental tools, and the translation of model-system insights into crop-relevant contexts. These contributions also show that Arabidopsis research remains highly dynamic. It continues not only to reveal fundamental biological mechanisms, but also to generate technologies, hypotheses, and comparative frameworks that benefit the broader plant science community.

We hope this Research Topic will serve as both a record of selected advances presented in connection with ICAR2024 and a stimulus for continued interdisciplinary collaboration. The Research Topic highlights the enduring value of Arabidopsis as a model system and its growing role as a bridge between mechanistic plant biology and agricultural innovation.

## References

[B1] Brady S. Auge G. Ayalew M. Balasubramanian S. Hamann T. Inzé D. . (2025). Arabidopsis research in 2030: Translating the computable Arabidopsis into crops and humans. Plant J. 121, e70047. doi: 10.1111/tpj.70047 40028766 PMC11874203

[B2] Koornneef M. Meinke D. (2010). The development of *Arabidopsis* as a model plant. Plant J. 61, 909–921. doi: 10.1111/j.1365-313X.2009.04086.x 20409266

[B3] Woodward A. W. Bartel B. (2018). Biology in bloom: A primer on the *Arabidopsis thaliana* model system. Genetics 208, 1337–1349. doi: 10.1534/genetics.118.300755 29618591 PMC5887134

[B4] Yaschenko A. E. Alonso J. M. Stepanova A. N. (2025). *Arabidopsis* as a model for translational research. Plant Cell 37, koae065. doi: 10.1093/plcell/koae065 38411602 PMC12082644

